# Influence of dietary L‐carnitine and lysine–methionine levels on reproductive performance and blood metabolic constituents of breeder ducks

**DOI:** 10.1111/rda.14047

**Published:** 2021-11-23

**Authors:** Maryam Mirzaei, Mehrdad Bouyeh, Afshin Zahedi, Alireza Seidavi, Rifat Ullah Khan, Vincenzo Tufarelli, Vito Laudadio, Mohamed E. Abd El‐Hack, Marco Ragni, Ayman E. Taha, Ayman A. Swelum

**Affiliations:** ^1^ Department of Animal Science Rasht Branch Islamic Azad University Rasht Iran; ^2^ Department of Veterinary Science Rasht Branch Islamic Azad University Rasht Iran; ^3^ College of Veterinary Sciences Faculty of Animal Husbandry & Veterinary Sciences The University of Agriculture Peshawar Pakistan; ^4^ Section of Veterinary Science and Animal Production Department of DETO University of Bari 'Aldo Moro' Valenzano, Bari Italy; ^5^ Poultry Department Faculty of Agriculture Zagazig University Sharkia Egypt; ^6^ Department of Agro‐Environmental and Territorial Sciences University of Bari ‘Aldo Moro’ Bari Italy; ^7^ Department of Animal Husbandry and Animal Wealth Development Faculty of Veterinary Medicine Alexandria University Edfina Egypt; ^8^ Department of Theriogenology Faculty of Veterinary Medicine Zagazig University Sharkia Egypt

**Keywords:** carnitine, duck, essential amino acids, reproduction, serum metabolites

## Abstract

The present study aimed to evaluate the influence of dietary supplementation of different levels of L‐carnitine and/or lysine–methionine (Lys‐Met) on reproductive performance of breeder ducks. Three L‐carnitine (0, 75 and 150 mg/kg) and three lysine–methionine (100%, 110% and 120% above the NRC (*Nutrient requirements of poultry*, 1994, National Academy Press) recommendations) levels were fed to 180 breeder ducks (144 females and 36 males) in a completely randomized design for 49 days. Laying performance and reproductive traits were evaluated; additionally, uric acid, total protein total, triglycerides, total cholesterol, low‐density lipoprotein, high‐density lipoprotein, aspartate aminotransferase (AST) and alanine aminotransferase (ALT) were assessed. The Lys‐Met above 100% NRC (*Nutrient requirements of poultry*, 1994, National Academy Press) recommendations with or without L‐carnitine improved feed utilization (*p* < .05). Furthermore, Lys‐Met above 100% recommendations without L‐carnitine improved egg fertility and hatchability. Fertility and hatchability improved in breeders fed on L‐carnitine with 120% Lys‐Met (*p* < .05). Serum glucose increased and total cholesterol reduced on 100% Ly‐Met without L‐carnitine or 110% Ly‐Met with 150 mg L‐carnitine (*p* < .05). Glucose was reduced, while total cholesterol increased on 75 mg L‐carnitine and 100% Lys‐Met (*p* < .05). Increasing Lys‐Met without L‐carnitine reduced serum protein (*p* < .05). Albumin and ALT increased on 75 mg L‐carnitine–100% Lys‐Met and reduced on 150 mg L‐carnitine–120% Lys‐Met (*p* < .05). There were no interaction effects on globulin, uric acid and AST (*p* > .05). Thus, based on findings, breeder ducks responded to dietary Lys‐Met more efficiently than L‐carnitine; however, more research is needed to evaluate also economic aspects related to L‐carnitine dietary supplementation.

## 1 INTRODUCTION

Although duck meat is becoming popular (Abd El‐Hack et al., 2019a; 2019b; Abo Ghanima, Abd El‐Aziz, et al., 2020; Abo Ghanima, Abd El‐Hack, et al., 2020; Farghly et al., 2018, 2019), the high fat content compared with other poultry species limits its acceptability by most consumers. Several dietary manipulations including addition of amino acids and metabolic intermediates are known to reduce abdominal fat and improve lean muscle depositions (Castro & Kim, 2020; Wu et al., 2011; Wu et al., 2021). Increasing dietary L‐methionine concentration significantly reduced abdominal fat content in ducks (Rehman et al., 2019; Xie et al., 2006), geese (Ashour et al., 2020; Wang et al., 2010) and broiler chickens (Andi, 2012). Moreover, Grisoni et al. (1991) and Attia (2003) reported significant reductions in abdominal fat and increased lean meat deposition with lysine supplementation in broiler chickens and ducks, respectively. These amino acids act mainly by suppressing lipogenesis through inactivation of fatty acid synthase and promotion of lipolysis by stimulating the secretion of hormone‐sensitive lipase (Alagawany et al., 2016; Reda et al., 2015; Takahashi & Akiba, 1995).

L‐carnitine is the biological active form of carnitine, synthesized endogenously from lysine and methionine. L‐carnitine regulates lipid and energy metabolism (Rehman, Chand, et al., 2017; Rehman, Naz, et al., 2017) by promoting mitochondrial β‐oxidation of long‐chain fatty acids (Abd El‐Wahab et al., 2015). Studies have shown that exogenous carnitine reduces the concentration of plasma, very‐low‐density lipoprotein cholesterol (VLDL‐C) and VLDL triglycerides (TG) in hyperlipidaemic rabbits and plasma lipoprotein levels in type 2 diabetic patients with hypercholesterolaemia (Rajasekar et al., 2005; Rehman et al., 2018). Plasma glucose concentration increased with carnitine supplementation due to increased fatty acid oxidation and subsequent decreased oxidation of gluconeogenic precursors (Greenwood et al., 2001). However, poultry have limited ability to synthesize carnitine in diets low in lysine and methionine. This has increased research interest in L‐carnitine supplementation of poultry diets. Supplementing diets with L‐carnitine was found to reduce carcass fat deposition in ducks (Arslan, 2006), but available reports on the effects on reproductive performance and blood metabolites of this species are still limited. Therefore, the present study investigated the effects of different dietary levels of L‐carnitine and lysine–methionine (Lys‐Met) on some reproductive traits and blood constituents of native breeder ducks.

## 2 MATERIALS AND METHODS

All the experimental procedures were carried out according to the approved protocols by the Institutional Animal Care and Use Committee (IACUC) of the Institutions, and care was followed to minimize the number of animals used.

### 2.1 Animals, management and treatments

The experiment was conducted at the Native Duck Rearing and Breeding Center, Fuman, Iran, and Rasht Branch, Islamic Azad University, Rasht, Iran. A total of 180 (144 ♀ and 36 ♂) native breeder Guilan ducks were weighed individually (1,740 ± 42.3 g) and divided into 36 groups of five birds (four ♀ and one ♂). Ducks were fed an isocaloric and isonitrogenous basal diet (Table 1) with different levels of L‐carnitine and lysine–methionine (Lys‐Met) in a 3 × 3 factorial experiment laid in a completely randomized design for 49 days.

**TABLE 1:** Ingredient composition and calculated analysis of the experimental basal diet

Ingredients	g/kg (as‐fed basis)
Corn	570.0
Soya bean meal (44% CP)	195.4
Wheat bran	125.0
Alfalfa meal	50.0
Oyster shell	39.3
Dicalcium phosphate	12.0
Vitamin–mineral premix^a^	4.0
Salt	2.5
Lysine hydrochloride	1.0
DL‐methionine	0.8
Calculated nutrients	
Metabolizable energy (kcal/kg diet)	2,561
Crude protein	163.0
Crude fibre	46.0
Linoleic acid	16.0
Calcium (g/kg)	19.0
Available phosphorus	3.7
Methionine	3.3
Lysine	8.6

^a^ Provide per kg diet: vitamin A: 5,000 IU/g; vitamin D3: 500 IU/g; vitamin E: 3 mg/g; vitamin K3: 1.5 mg/g; vitamin B2: 1 mg/g; calcium pantothenate: 4 mg/g; NIACIN: 15 mg/g; vitamin B6: 13 mg/g; Cu: 3 mg/g; Zn: 15 mg/g; Mn: 20 mg/g; Fe: 10 mg/g; K: 0.3 mg/g.

Their dietary treatments were as follows:

1. L‐carnitine (0 mg/kg) and Lys‐Met (100% recommendations of NRC, 1994).
2. L‐carnitine (75 mg/kg) and Lys‐Met (100% recommendations of NRC, 1994).
3. L‐carnitine (150 mg/kg) and Lys‐Met (100% recommendations of NRC, 1994).
4. L‐carnitine (0 mg/kg) and Lys‐Met (110% recommendations of NRC, 1994).
5. L‐carnitine (75 mg/kg) and Lys‐Met (110% recommendations of NRC, 1994).
6. L‐carnitine (150 mg/kg) and Lys‐Met (110% recommendations of NRC, 1994).
7. L‐carnitine (0 mg/kg) and Lys‐Met (120% recommendations of NRC, 1994).
8. L‐carnitine (75 mg/kg) and Lys‐Met (120% recommendations of NRC, 1994).
9. L‐carnitine (150 mg/kg) and Lys‐Met (120% recommendations of NRC, 1994).

Birds were housed in floor pens measuring 200 × 200 cm^2^. The house temperature and relative humidity were maintained at 13°C and 55%–65%, respectively, throughout the experimental period. Feed and water were provided ad libitum during the experimental period. The lighting programme was 16 hr of light and 8 hr of dark throughout the duration of the study.

### 2.2 Laying performance and reproductive traits

Feed intake was monitored per pen as the difference between the quantities of fed and leftover. Eggs were recorded per pen, and percentage egg production was calculated as 100 × (eggs collected ÷ hens in the pen). All eggs produced were weighed using a digital portable scale (Jadever JKH‐500 series; SmartFox) sensitive to ±0.1 g, and the mean egg weight was recorded per pen. Feed conversion ratio (FCR) was calculated as g feed consumed to g egg produced.

Eggs collected were labelled per replicate and stored at 17°C and 74% relative humidity for 1 week. Eggs (68.7 ± 1.8 g) were candled and fertile ones incubated in the setter at 37.5°C and 60% humidity with regular turning for the first 25 days. On Day 25, eggs were transferred to the hatcher operated at 37°C and 71% relative humidity. At the end of 28 days of incubation, ducklings were removed and counted. Fertility and hatchability parameters were calculated as:

Fertility(%)=(numberoffertileeggs/numberoflaideggs)×100.

Hatchabilityoffertileeggs(%)=(numberofhatchedeggs/numberoffertileeggs)×100.

### 2.3 Blood biochemical constituents

On Day 49 of the trial, three representative birds per replicate were selected and blood samples (1 ml/bird) collected, from the wing vein, for biochemical analysis according to Jahanpour et al. (2013). Blood samples were transferred from the syringe into sample tubes coated with 10 mg of ethylenediaminetetraacetic acid (EDTA) and centrifuged at 1000 g × 20 min to ensure separation of the blood cells from the plasma. Plasma constituents were analysed following the standard protocols using the Roche Cobas Integra 400 Plus autoanalyzer (Roche Diagnostics GmbH). Uric acid, total protein total, TG, cholesterol, low‐density lipoprotein (LDL), high‐density lipoprotein (HDL), aspartate aminotransferase (AST) and alanine aminotransferase (ALT) were assayed using commercial kits (Teif Azmoon Pars, Co.).

### 2.4 Statistical analysis

Data were analysed using the GLM procedure of SAS software (SAS, 2000). The model included L‐carnitine and Lys‐Met as main effects. The interaction among main effects was included in the model. Means were compared using Duncan’s multiple range test and significance levels reported at 5% probability. The model used was as follows: *Y_ijk_* = *μ* + *A_i_* + *B_j_* + *AB_ij_* + *e_ijk_*, where *μ* = the common mean, *A_i_* = the effects of the L‐carnitine, *B_j_* = the effect of the Lys‐Met, AB_ij =_ the effect of the *i*th A with the *j*th B, and *e_ijk_* = the random error. Before performing the statistical analysis, all data were tested by the normality test and transformed, if necessary.

## 3 RESULTS

### 3.1 Laying performance and reproductive traits

The results of laying performance and reproductive traits of the ducks (Table 2) showed no interaction effects on feed intake, hen‐day production, egg weight and egg mass of the ducks (*p* > .05). Dietary Lys‐Met above 100% of NRC (1994) recommendations with or without L‐carnitine improved FCR (feed: gain) of ducks (*p* < .05). In the main effects, neither L‐carnitine nor Lys‐Met level affected the performance parameters observed (*p* > .05).

**TABLE 2:** Productive and reproductive performance of ducks fed different levels of dietary L‐carnitine and lysine–methionine

Treatments		Feed intake (kg/5 ducks)	Egg weight (g)	Egg mass (kg/4 ducks)	Hen‐day production (%)	FCR	Fertility (%)	Hatchability (%)
Effect of adding different levels of L‐Carnitine (mg/kg)								
L‐Carnitine	0	8.1	69	2.1	15.7	3.9	24.91	15.97
	75	7.7	68.7	2.5	18.5	3.1	26.55	17.27
	150	8.1	69.7	2.7	18.1	3	21.80	17.55
*p*‐Value		.481	.571	.309	.419	.323	.094	.088
Effect of adding different levels of Lys‐Met (%)								
Lys‐Met	100	8.1	70.1	1.5	10.7	5.4	10.44^c^	2.22^c^
	110	7.9	68.5	2.3	16.8	3.4	19.08^b^	13.58^b^
	120	7.9	68.8	3.6	24.7	2.2	43.75^a^	35.00^a^
*p*‐Value		.243	.474	.391	.712	.233	.021	.017
Effect of interaction between adding different levels of L‐Carnitine and Lys‐Met								
L‐Carnitine	Lys‐Met							
0	100	9.3	70.1	1.1	8	8.5^a^	10^c^	1.7^c^
	110	7.8	67.8	1.8	13.9	4.3^b^	21^ab^	21.3^ab^
	120	7.4	69.1	3.4	25.1	2.2^de^	43.8^a^	25^ab^
75	100	7.3	70.7	1.7	12.5	4.3^b^	14.7^b^	1.3^c^
	110	7.8	68.1	2.6	19.8	3^cd^	20^b^	10.5^b^
	120	8	67.4	3.1	23.2	2.6^cd^	45^a^	40^a^
150	100	7.7	69.5	1.6	11.6	4.8^b^	6.7^c^	3.7^c^
	110	8.1	69.6	2.3	16.8	3.5^bc^	16.3^b^	9^b^
	120	8.4	69.9	4.2	25.8	2^e^	42.5^a^	40^a^
*SEM*		0.350	1.18	0.112	1.61	0.341	5.603	1.078
*p*‐Value		.117	.521	.673	.484	.041	.014	.039

Note

Mean values with different superscripts within the same column differ significantly (*p* < .05).

Abbreviation: *SEM*, standard error of the mean.

Egg fertility and hatchability indexes were significantly influenced by Lys‐Met and Lys‐Met interaction (*p* < .05). Feeding of Lys‐Met above 100% of NRC recommendations without L‐carnitine improved egg fertility and hatchability, but the addition of L‐carnitine (75 or 150 mg) improved fertility and hatchability only when Lys‐Met was increased to 120% of the NRC recommendations (*p* < .05). Based on the main effects, L‐carnitine addition did not affect the reproductive parameters (*p* > .05), but these were improved with increasing Lys‐Met above 100% of NRC recommendations (*p* < .05).

### 3.2 Metabolic parameters

The results of blood energy and protein metabolism parameters are presented in Tables 3 and 4, respectively. Serum glucose was increased and total cholesterol reduced by supplementing Lys‐Met at 100% without L‐carnitine or 110% with 150 mg L‐carnitine (*p* < .05). Feeding of diet with 75 mg L‐carnitine and 100% Lys‐Met reduced glucose and increased cholesterol (*p* <.05). There was no interaction effect on other energy blood metabolism parameters studied (*p* >.05). In the main effects, L‐carnitine did not affect any of the energy metabolism parameters (*p* > .05), but Lys‐Met level affected serum LDL content (*p* < .05). Increasing Lys‐Met level from 100% to 110% reduced LDL (*p* < .05), but by a further increase in Lys‐Met level to 120%, the decrease in LDL was not differed significantly compared with Lys‐Met level at 100% (*p* > .05). L‐carnitine or Lys‐Met had no effects on other energy metabolism parameters assessed (*p* > .05). Increasing Lys‐Met without L‐carnitine reduced serum total protein (*p* < .05), but this was restored with L‐carnitine addition. Higher serum albumin and ALT levels were found on ducks fed 75 mg L‐carnitine with 100% Lys‐Met and lower values on 150 mg L‐carnitine with 120% Lys‐Met (*p* < .05). There were no interaction effects on serum globulin, uric acid and AST (*p* > .05).

**TABLE 3:** Energy metabolic parameters of ducks fed different levels of dietary L‐carnitine and lysine–methionine

Treatments		Glucose (mg/dl)	Total cholesterol (mg/dl)	Triglycerides (mg/dl)	HDL (mg/dl)	LDL (mg/dl)	HDL/LDL
Effect of adding different levels of L‐carnitine (mg/kg)							
L‐Carnitine	0	170.4	184.3	356.2	56.5	71.2	1.8
	75	166.2	220.8	418.4	57	83.8	0.9
	150	175.8	198.9	367	36.3	73.4	1.8
*p*‐Value		.418	.558	.931	.519	.929	.450
Effect of adding different levels of Lys‐Met (%)							
Lys‐Met	100	170.8	229.5	630.9	56.5	126.3^a^	0.2
	110	164.8	183	230.1	65.8	46^b^	2.2
	120	176.7	191.4	280.5	57.5	56.2^ab^	2.1
*p*‐Value		.300	.352	.062	.149	.021	.361
Effect of interaction between adding different levels of L‐carnitine and Lys‐Met							
L‐Carnitine	Lys‐Met						
0	100	189.5^a^	144.5^b^	606	45.8	81	0.5
	110	136^c^	224.3^ab^	99	88	69.8	1.1
	120	185.7^a^	184^b^	363.5	55.8	72.8	0.8
75	100	151.3^bc^	328.3^a^	623	46.3	94.75	0.3
	110	175.00^ab^	171.58^b^	218.0	82.25	73.8	0.9
	120	172.3^ab^	162.5^b^	414.3	52.5	83	0.6
150	100	171.8^ab^	215.8^ab^	663.8	47.5	93	0.4
	10	183.3^a^	153.3^b^	373.3	47.3	74.5	0.4
	120	172.3^ab^	227.8^ab^	63.8	64.3	72.8	1.2
*SEM*		8.73	41.19	215.03	24.70	42.98	0.30
*p*‐Value		.019	.01	.409	.484	.408	.440

Note

Mean values with different superscripts within the same column differ significantly (*p* <.05).

Abbreviation: *SEM*, standard error of the mean.

**TABLE 4:** Protein metabolic parameters and hepatic functionality of ducks fed different levels of dietary L‐carnitine and lysine–methionine

Treatments		Total protein (g/dl)	Albumin (g/dl)	Globulin (g/dl)	Uric acid (mg/dl)	AST (U/L)	ALT (U/L)
Effect of adding different levels of L‐carnitine (mg/kg)							
L‐Carnitine	0	5.2^b^	2.7	2.5	7.8	39.1	33.9
	75	5.8^a^	3.2	2.6	9.2	37.1	37
	150	5.3^ab^	2.8	2.4	9.3	47.1	42.8
*p*‐Values		.028	.275	.969	.498	.706	.378
Effect of adding different levels of Lys‐Met (%)							
Lys‐Met	100	5.7	3.3^a^	2.4	8	51.9	37.4
	110	5.3	2.9^ab^	2.4	9	37.3	34.1
	120	5.3	2.3^b^	2.7	9.23	34	32.3
*p*‐Values		.169	.011	.752	.515	.334	.159
Effect of interaction between adding different levels of L‐carnitine and Lys‐Met							
L‐Carnitine	Lys‐Met						
0	100	5.7^abc^	3.2^ab^	2.5	6.2	42.5	41.5^ab^
	110	5.2^c^	2.4^ab^	2.7	9.2^a^	45	29.7^b^
	120	4.7^c^	2.4^ab^	2.3	8	30.8	33.5^ab^
75	100	6.3^a^	3.8^a^	2.5	8.5	36.8	39.8^a^
	110	5.2^abc^	2.7^ab^	2.5	10.5	40.5	36.8^ab^
	120	5.8^ab^	3.2^ab^	2.6	8.7	34	34.5^ab^
150	100	5.1^abc^	2.9^ab^	2.1	9.4	36.5	91^a^
	110	5.4^abc^	3.4^ab^	2	7.3	36.5	38.8^ab^
	120	5.3^abc^	2.1^b^	3.2	11.2	38.3	29.8^b^
*SEM*		0.44	0.40	0.57	1.27	15.45	17.10
*p*‐Values		.009	.015	.934	.350	.541	.015

Note

Mean values with different superscripts within the same column differ significantly (*p* < .05).

Abbreviation: *SEM*, standard error of the mean.

## 4 DISCUSSION

### 4.1 Laying performance and reproductive traits

The effects of dietary supplemental L‐carnitine, Lys and Met on poultry performance have been inconsistent. In fact, while some studies have reported improved performance, others found no significant effect. In the present study, increasing Lys‐Met above NRC recommendations with or without L‐carnitine improved feed efficiency, but L‐carnitine or Lys‐Met alone had no effects on feed utilization and egg production. These results partially agreed with those obtained by El‐kelawy, 2017; Rizk et al., 2019; Sigolo et al., 2019. In a previous study, Leibetseder (1995) also found no effect of dietary L‐carnitine (500 mg/kg) on laying performance of broiler breeders. These data might imply that the effects of L‐carnitine and Lys‐Met are additive rather than antagonistic. Both Lys and Met are essential for the synthesis of egg and body protein, and a shortage in either of these amino acids leads to a reduction in egg production (Hiramoto et al., 1990). L‐carnitine, which is mainly biosynthesized from Lys and Met (Abd El‐Wahab et al., 2015; Arslan, 2006), regulates β‐oxidation of fatty acids and prevents oxidative reactions by forming a defence line against reactive oxygen species (Arenas et al., 1998). A sufficient supply of L‐carnitine in the diet is therefore essential to provide for these functions and preserve Lys and Met from conversion to carnitine, making them available for tissue synthesis. Supplementation of low protein diets with carnitine in broiler chickens did promote growth in animals through its methionine‐sparing effect (Golrokh et al., 2016; Panahi et al., 2019; Tufarelli et al., 2020). Owen et al. (1996) also reported sparing of branched‐chain amino acids from oxidation in tissues with an increased supply of carnitine.

Improving egg fertility and hatchability can be achieved by increasing Lys‐Met alone above 100% of NRC recommendations or by the addition of L‐carnitine. This pattern further confirms the sparing effect of these amino acids from endogenous biosynthesis of carnitine. The beneficial effects of L‐carnitine on poultry fertility are documented. L‐carnitine increases sperm quantity and quality by protecting sperm against per‐oxidative damage and dysfunction (Alvarez & Storey, 1989). Active transporters in the epididymal lumen release circulating L‐carnitine into the lumen, which is controlled by androgens and delivered into spermatozoa, where it remains (Cooper, Gudermann, et al., 1986; Cooper, Yeung, et al., 1986; Enomoto et al., 2002; Jeulin & Lewin, 1996; Kobayashi et al., 2005). This coupled with the action of L‐carnitine on energy metabolism at cellular level, and its inhibition of apoptosis (Agarwal & Said, 2004; Balercia et al., 2005; Lenzi et al., 2003, 2004) could possibly explain its effects on reproductive cells. Ad libitum consumption of diets containing L‐carnitine at levels of 500 mg/kg (Neuman et al., 2002) or 125 mg/kg (Zhai et al., 2007) increased sperm concentration and reduced sperm lipid peroxidation in white leghorn roosters. Sarica et al. (2007) also reported a significant increase in sperm viability of mature male Japanese quail breeders supplemented with 250 or 500 mg L‐carnitine/kg diet. L‐carnitine supplementation also increased semen quantity and quality in broiler breeders (Golzar et al., 2007) and ostriches (Adabi et al., 2008). Several other authors (Leibetseder, 1995; Neuman et al., 2002; Rabie et al., 1997; Suchý et al., 2008) have also confirmed the beneficial effect of L‐carnitine on poultry reproduction. Dietary sulphur amino acid levels also improve egg quality (Narvaez‐Solarte, 1996), which influence egg storage and hatchability (Abd El‐Hack et al., 2019a; 2019b; Toro et al., 2015).

### 4.2 Metabolic parameters

We could not explain the pattern of energy and protein metabolism in this study, but a possible increase in fatty acid oxidation and the subsequently reduced oxidation of gluconeogenic precursors (Greenwood et al., 2001) could be a possible speculation. The available findings on Lys and Met supplementation alone or in combination with L‐carnitine on serum biochemical constituents in poultry are inconsistent. In opposition to our results, Arslan et al. (2004), Taraz and Dastar (2008) and Babak et al. (2015) found no effects of dietary levels of L‐carnitine and Lys‐Met on serum glucose and triglyceride concentrations in broiler chickens. Zhai et al. (2016) found no effects of Lys or Met levels on serum metabolites of male broilers from 21 to 42 days of age. Several factors including species of bird, age, diet composition and concentration of amino acids and carnitine may influence the response of poultry to Lys‐Met and L‐carnitine supplementation with regard to serum composition. The peculiarities in fatty acid composition of duck muscle (Ali et al., 2007) may also be implicated in the differences in serum composition observed between this study and those found in other poultry species.

## 5 CONCLUSION

It appears from the obtained findings that breeding ducks respond to Lys‐Met more efficiently than L‐carnitine. Increasing dietary Lys‐Met above NRC recommendations has no effect on egg production but improved feed utilization, egg fertility and hatchability. The pattern of the hens’ response in terms of serum metabolites is not consistent, and this needs further investigations. Because carnitine is biosynthesized endogenously from Lys‐Met, the supply of L‐carnitine in the diet will spare these essential amino acids. However, L‐carnitine is still a relatively expensive additive in many countries and there is a need to validate the cost‐effectiveness of its dietary inclusion. However, their availability, ease of use and their positive impact on ducks may make their use in duck feeds justified.

## CONFLICT OF INTEREST

None of the authors have any conflict of interest to declare.

## AUTHOR CONTRIBUTIONS

Maryam Mirzaei, Mehrdad Bouyeh, Afshin Zahedi and Alireza Seidavi conceptualized the study, contributed to methodology, validation and investigation, performed formal analysis and wrote the original draft. Alireza Seidavi, Rifat Ullah Khan, Vincenzo Tufarelli, Mohamed E. Abd El‐Hack, Ayman E. Taha and Ayman A Swelum performed investigation and data curation. Alireza Seidavi, Vincenzo Tufarelli, Marco Ragni, Vito Laudadio and Ayman A Swelum provided resources, performed funding acquisition and supervision, and wrote, reviewed and edited the manuscript.

## Data Availability

The data that support the findings of this study are available upon request from the corresponding author.

## References

[rda14047-bib-0001] Abd El‐Hack, M. E. , Hurtado, C. B. , Toro, D. M. , Alagawany, M. , Abdelfattah, E. M. , & Elnesr, S. S. (2019a). Fertility and hatchability in duck eggs. World's Poultry Science Journal, 75(4), 599–608. 10.1017/S0043933919000060

[rda14047-bib-0002] Abd El‐Hack, M. E. , Hurtado, C. B. , Toro, D. M. , Alagawany, M. , Abdelfattah, E. M. , & Elnesr, S. S. (2019b). Impact of environmental and incubation factors on hatchability of duck eggs. Biological Rhythm Research, 1–10. 10.1080/09291016.2019.1628394

[rda14047-bib-0003] Abd El‐Wahab, A. , Aziza, A. , & El‐Adl, M. (2015). Impact of dietary excess methionine and lysine with or without addition of L‐carnitine on performance, blood lipid profile and litter quality in broilers. Asian Journal of Animal and Veterinary Advances, 10(5), 191–202.

[rda14047-bib-0004] Abo Ghanima, M. M. , Abd El‐Aziz, A. , El‐Edel, M. , Ashour, E. A. , Abd El‐Hack, M. E. , Othman, S. , Alqaraawi, M. , Allam, A. , & Khafaga, A. (2020). The influences of various housing systems on growth, carcass traits, meat quality, immunity and oxidative stress of meat‐type ducks. Animals, 10(3), 410. 10.3390/ani10030410 PMC7143679 32121623

[rda14047-bib-0005] Abo Ghanima, M. M. , Abd El‐Hack, M. E. , Mohamed, E. , Taha, A. E. , Tufarelli, V. , Laudadio, V. , & Naiel, M. A. (2020). Assessment of stocking rate and housing system on performance, carcass traits, blood indices, and meat quality of french pekin ducks. Agriculture, 10(7), 273. 10.3390/agriculture10070273

[rda14047-bib-0006] Adabi, S. H. G. , Hajibabaei, A. , Farahvash, T. , Lotfollahian, H. , & Moslemi, P. F. (2008). L‐carnitine effect on quantity and quality of African black neck ostrich sperm. Journal of Animal and Veterinary Advances, 3, 369–374. 10.3923/ajava.2008.369.374

[rda14047-bib-0007] Agarwal, A. , & Said, T. M. (2004). L‐Carnitines and male infertility. Reproductive Biomedicine Online, 8, 376–384. 10.1016/S1472-6483(10)60920-0 15149558

[rda14047-bib-0008] Alagawany, M. , Abd El‐Hack, M. E. , Arif, M. , & Ashour, E. A. (2016). Individual and combined effects of crude protein, methionine, and probiotic levels on laying hen productive performance and nitrogen pollution in the manure. Environmental Science and Pollution Research, 23(22), 22906–22913. 10.1007/s11356-016-7511-6 27572695

[rda14047-bib-0009] Ali, M. S. , Kang, G. H. , Yang, H. S. , Jeong, J. Y. , Hwang, Y. H. , Park, G. B. , & Joo, S. T. (2007). A comparison of meat characteristics between duck and chicken breast. Asian‐Australasian Journal of Animal Science, 20(6), 1002–1006. 10.5713/ajas.2007.1002

[rda14047-bib-0010] Alvarez, J. G. , & Storey, B. T. (1989). Role of glutathione peroxidase in protecting mammalian spermatozoa from loss of motility caused by spontaneous lipid peroxidation. Gamete Research, 23, 77–90. 10.1002/mrd.1120230108 2545584

[rda14047-bib-0011] Andi, M. A. (2012). Effects of additional DL‐methionine in broiler starter diet on blood lipids and abdominal fat. African Journal of Biotechnology, 11, 7579–7581.

[rda14047-bib-0012] Arenas, J. , Rubio, J. C. , Martin, M. A. , & Campos, Y. (1998). Biological roles of L‐carnitine in perinatal metabolism. Early Human Development, 53, 43–50. 10.1016/s0378-3782(98)00064-4 10102654

[rda14047-bib-0013] Arslan, C. (2006). L‐Carnitine and its use as feed additive in poultry feeding a review. Revue de Médecine. Vétérinaire, 157, 134–142.

[rda14047-bib-0014] Arslan, C. , Citil, M. , & Saatci, M. (2004). Effects of L‐carnitine administration on growth performance, carcass traits, serum lipids and abdominal fatty acid composition of geese. Revue de Médecine. Vétérinaire, 155, 315–320. 10.1080/00039420310001607734 14620911

[rda14047-bib-0015] Ashour, E. A. , Abou‐Kassem, D. E. , Abd El‐Hack, M. E. , & Alagawany, M. (2020). Effect of dietary protein and TSAA levels on performance, carcass traits, meat composition and some blood components of Egyptian geese during the rearing period. Animals, 10(4), 549. 10.3390/ani10040549 PMC7222406 32218190

[rda14047-bib-0016] Attia, Y. A. (2003). Performance, carcass characteristics, meat quality and plasma constituents of meat type drakes fed diets containing different levels of lysine with or without a microbial phytase. Archives of Animal Nutrition, 57, 39–48. 10.1080/0003942031000086635 12801078

[rda14047-bib-0017] Babak, H. , Dadashbeiki, M. , Bouyeh, M. , Seidavi, A. , van den Hoven, R. , & Gamboa, S. (2015). Effect of different levels of L‐carnitine and lysine‐methionine on broiler blood parameters. Revista. MVZ Cordoba, 20(3), 4698–4708.

[rda14047-bib-0018] Balercia, G. , Regoli, F. , Armeni, T. , Koverech, A. , Mantero, F. , & Boscaro, M. (2005). Placebo‐controlled double‐blind randomized trial on the use of L‐carnitine, l‐acetylcarnitine, or combined L‐carnitine and ‐acetylcarnitine in men with idiopathic asthenozoospermia. Fertility and Sterility, 84, 662–671. 16169400 10.1016/j.fertnstert.2005.03.064

[rda14047-bib-0019] Castro, F. L. D. S. , & Kim, W. K. (2020). Secondary functions of arginine and sulfur amino acids in poultry health. Animals, 10(11), 2106. 10.3390/ani10112106 PMC7697735 33202808

[rda14047-bib-0020] Cooper, T. G. , Gudermann, T. W. , & Yeung, C. H. (1986). Characteristics of the transport of carnitine into the cauda epididymis of the rat as ascertained by luminal perfusion in vitro. International Journal of Andrology, 9, 348–358. 3570531 10.1111/j.1365-2605.1986.tb00897.x

[rda14047-bib-0021] Cooper, T. G. , Yeung, C. H. , & Weinbauer, G. F. (1986). Transport of carnitine by the epididymis of the cynomolgus macaque (*Macaca fascicularis*). Journal of Reproduction and Fertility, 77, 297–301. 10.1530/jrf.0.0770297 3723474

[rda14047-bib-0022] El‐kelawy, M. (2017). Effects of L‐carnitine on production performance, blood parameters, lipid metabolism and antioxidative properties of broiler chicks. Egyptian Poultry Science Journal, 37(3), 873–892. 10.21608/epsj.2017.7737

[rda14047-bib-0023] Enomoto, A. , Wempe, M. F. , Tsuchida, H. , Shin, H. J. , Cha, S. H. , Anzai, N. , Goto, A. , Sakamoto, A. , Niwa, T. , Kanai, Y. , Anders, M. W. , & Endou, H. (2002). Molecular identification of a novel carnitine transporter specific to human testis. The Journal of Biological Chemistry, 277, 36262–36271. 10.1074/jbc.M203883200 12089149

[rda14047-bib-0024] Farghly, M. F. A. , Abd El‐Hack, M. E. , Alagawany, M. , Saadeldin, I. M. , & Swelum, A. A. (2018). Wet feed and cold water as heat stress modulators in growing Muscovy ducklings. Poultry Science, 97(5), 1588–1594. 10.3382/ps/pey006 29462361

[rda14047-bib-0025] Farghly, M. F. , Abd El‐Hack, M. E. , Alagawany, M. , Saadeldin, I. M. , & Swelum, A. A. (2019). Ameliorating deleterious effects of heat stress on growing Muscovy ducklings using feed withdrawal and cold water. Poultry Science, 98(1), 251–259. 10.3382/ps/pey396 30289496

[rda14047-bib-0026] Golrokh, A. J. , Bouyeh, M. , Seidavi, A. , van den Hoven, R. , Laudadio, V. , & Tufarelli, V. (2016). Effect of different dietary levels of atorvastatin and L‐carnitine on performance, carcass characteristics and plasma constitutes of broiler chickens. The Journal of Poultry Science, 53(3), 201–207. 10.2141/jpsa.0150184 PMC7477135 32908384

[rda14047-bib-0027] Golzar, A. S. H. , Rahimi, S. H. , Kamali, M. A. , & Karimi‐Torshizi, M. A. (2007). The effects of two dietary levels of L‐carnitine and vegetable fat powder on quality of cockerels sperm, and fertility and hatchability in broiler breeders. Journal of Veterinary Research, 62, 107–114.

[rda14047-bib-0028] Greenwood, R. H. , Titgemeyer, E. C. , Stokka, G. L. , Drouillard, J. S. , & Loest, C. A. (2001). Effect of L‐carnitine on nitrogen retention and blood metabolites of growing steers and performance of finishing steers. Journal of Animal Science, 79, 254–260. 11204708 10.2527/2001.791254x

[rda14047-bib-0029] Grisoni, M. L. , Uzu, G. , Larbier, M. , & Geraert, P. A. (1991). Effect of dietary lysine on lipogenesis in broilers. Reproduction Nutrition Development, 31, 683–690. 1777060 10.1051/rnd:19910608

[rda14047-bib-0030] Hiramoto, K. , Muramatsu, T. , & Okumura, J. (1990). Effect of methionine and lysine deficiencies on protein synthesis in the liver and oviduct and in the whole body of laying hens. Poultry Science, 69, 84–89. 10.3382/ps.0690084 2108438

[rda14047-bib-0031] Jahanpour, H. , Seidavi, A. R. , Qotbi, A. A. A. , & Payan‐Carreira, R. (2013). Effects of two levels of quantitative feed restriction for a 7‐ or 14‐ days period on broilers blood parameters. Acta Scientiae Veterinariae, 41, 1–11.

[rda14047-bib-0032] Jeulin, C. , & Lewin, L. M. (1996). Role of free L‐carnitine and acetyl‐L‐carnitine in post‐gonadal maturation of mammalian spermatozoa. Human Reproduction Update, 2, 87–102. 10.1093/humupd/2.2.87 9079406

[rda14047-bib-0033] Kobayashi, D. , Irokawa, M. , Maeda, T. , Tsuji, A. , & Tamai, I. (2005). Carnitine/organic cation transporter OCTN2‐mediated transport of carnitine in primary‐cultured epididymal epithelial cells. Reproduction, 130, 931–937. 10.1530/rep.1.00737 16322553

[rda14047-bib-0034] Leibetseder, J. (1995). Untersuchungen uber die Wirkungen von l‐Carnitin beim Huhn. Archives of Animal Nutrition, 48, 97–108. 8526737 10.1080/17450399509381832

[rda14047-bib-0035] Lenzi, A. , Lombardo, F. , Sgro, P. , Salacone, P. , Caponecchia, L. , Dondero, F. , & Gandini, L. (2003). Use of carnitine therapy in selected cases of male factor infertility: A double‐blind crossover trial. Fertility and Sterility, 79, 292–300. 10.1016/S0015-0282(02)04679-4 12568837

[rda14047-bib-0036] Lenzi, A. , Sgro, P. , Salacone, P. , Paoli, D. , Gilio, B. , Lombardo, F. , Santulli, M. , Agarwal, A. , & Gandini, L. (2004). A placebo‐controlled double‐blind randomized trial of the use of combined L‐carnitine and l‐acetyl‐carnitine treatment in men with asthenozoospermia. Fertility and Sterility, 81, 1578–1584. 10.1016/j.fertnstert.2003.10.034 15193480

[rda14047-bib-0037] Narvaez‐Solarte, W. V. (1996). Exigências em metionina+cistina para poedeiras leves e semipesadas & #91; dissertação & #93. Universidade Federal de Viçosa.

[rda14047-bib-0038] Neuman, S. L. , Lin, T. L. , & Hester, P. Y. (2002). The effect of dietary carnitine on semen traits of white leghorn roosters. Poultry Science, 81, 495–503. 10.1093/ps/81.4.495 11989749

[rda14047-bib-0039] NRC, National Research Council (1994). Nutrient requirements of poultry, 9th revised ed. National Academy Press.

[rda14047-bib-0040] Owen, K. Q. , Nelssen, J. L. , Goodband, R. D. , Weeden, T. L. , & Blum, S. A. (1996). Effect of L‐carnitine and soybean oil on growth performance and body composition of early‐weaned pigs. Journal of Animal Science, 74, 1612–1619. 10.2527/1996.7471612x 8818806

[rda14047-bib-0041] Panahi, H. , Bouyeh, M. , Behzadpour, D. , Seidavi, A. , Simões, J. , Tufarelli, V. , Staffa, V. N. , Tinelli, A. , Ayasan, T. , & Laudadio, V. (2019). Effect of dietary simvastatin and L‐carnitine supplementation on blood biochemical parameters, carcass characteristics and growth of broiler chickens. Journal of the Indonesian Tropical Animal Agriculture, 44(4), 372–381. 10.14710/jitaa.44.4.372-381

[rda14047-bib-0042] Rabie, M. H. , Szilagyi, M. , & Gippert, T. (1997). Effects of dietary L‐carnitine on the performance and egg quality of laying hens from 65–73 weeks of age. British Journal of Nutrition, 78, 615–623. 10.1079/BJN19970178 9389887

[rda14047-bib-0043] Rajasekar, P. , Katchapeswaran, R. M. , & Venkatraman, A. C. (2005). Intraperitoneal L‐carnitine regulates lipid metabolism and reduces oxidative stress in fructose‐induced hyperlipidemic rats. Diabetologia Croatica, 34, 87–96.

[rda14047-bib-0044] Reda, F. M. , Ashour, E. A. , Alagawany, M. , & Abd El‐Hack, M. E. (2015). Effects of dietary protein, energy and lysine intake on growth performance and carcass characteristics of growing Japanese quails (2015). *Asian* . Journal of Poultry Science, 9(3), 155–164. 10.3923/ajpsaj.2015.155.164

[rda14047-bib-0045] Rehman, A. U. , Arif, M. , Husnain, M. M. , Alagawany, M. , Abd El‐Hack, M. E. , Taha, A. E. , Elnesr, S. S. , Abdel‐Latif, M. A. , Othman, S. I. , & Allam, A. A. (2019). Growth performance of broilers as influenced by different levels and sources of methionine plus cysteine. Animal, 9(12), 1056. 10.3390/ani9121056 PMC6941102 31805723

[rda14047-bib-0046] Rehman, Z. , Chand, N. , & Khan, R. U. (2017a). The effect of vitamin E, L‐carnitine and ginger on production traits, immune response and antioxidant status in two broiler strains exposed to chronic heat stress. Environmental Science and Pollution Research, 24, 26851–26857. 10.1007/s11356-017-0304-8 28963619

[rda14047-bib-0047] Rehman, Z. , Chand, N. , Khan, R. U. , Naz, S. , & Alhidary, I. A. (2018). Serum biochemical profile of two broilers strains supplemented with vitamin E, raw ginger (*Zingiber officinale*) and L‐carnitine under high ambient temperatures. South African Journal of Animal Science, 48, 935–942.

[rda14047-bib-0048] Rehman, Z. , Naz, S. , Khan, R. U. , & Tahir, M. (2017b). An update on the potential application of L‐carnitine in poultry. World’s Poultry Science Journal, 73, 823–830.

[rda14047-bib-0049] Rizk, Y. S. , Fahim, H. N. , Beshara, M. M. , Mahrose, K. M. , & Awad, A. L. (2019). Response of duck breeders to dietary L‐Carnitine supplementation during summer season. Anais da Academia Brasileira de Ciências, 91(4), e20180907. 10.1590/0001-3765201920180907 31644644

[rda14047-bib-0050] Sarica, S. , Corduk, M. , Ensoy, U. , Basmacioglu, H. , & Karatas, U. (2007). Effects of dietary supplementation of L‐carnitine on performance, carcass and meat characteristics of quails. South African Journal of Animal Science, 37, 189–201. 10.4314/sajas.v37i3.4091

[rda14047-bib-0051] SAS Institute Inc (2000). SAS Online Doc. SAS Institute Inc.

[rda14047-bib-0052] Sigolo, S. , Deldar, E. , Seidavi, A. , Bouyeh, M. , Gallo, A. , & Prandini, A. (2019). Effects of dietary surpluses of methionine and lysine on growth performance, blood serum parameters, immune responses, and carcass traits of broilers. Journal of Applied Animal Research, 47(1), 146–153. 10.1080/09712119.2019.1583571

[rda14047-bib-0053] Suchý, P. , Straková, E. , & Vitula, F. (2008). The effect of a diet supplemented with l‐carnitine on egg production in pheasant (*Phasianus colchicus*). Czech Journal of Animal Science, 53(1), 31–35.

[rda14047-bib-0054] Takahashi, K. , & Akiba, Y. (1995). Effect of methionine supplementation on lipogenesis and lipolysis in broiler chicks. Japanese Poultry Science, 32(2), 99–106. 10.2141/jpsa.32.99

[rda14047-bib-0055] Taraz, Z. , & Dastar, A. (2008). Effect of L‐carnitine supplementing diets with different levels of protein on performance and blood parameters in broiler chickens. Journal of Agricultural Science and Natural Resources, 15(5), 114–122.

[rda14047-bib-0056] Toro, D. M. , Aguilar, Y. M. , Bertot, R. R. , Hurtado, C. B. , & Nava, O. R. (2015). Effect of dietary supplementation with *Morinda citrifolia* on productivity and egg quality of laying hens. Revista Ciencia y Agricultura, 12(2), 7–12. 10.19053/01228420.4348

[rda14047-bib-0057] Tufarelli, V. , Mehrzad‐Gilmalek, H. , Bouyeh, M. , Qotbi, A. , Amouei, H. , Seidavi, A. , Paz, E. , & Laudadio, V. (2020). Effect of different levels of L‐carnitine and excess lysine‐methionine on broiler performance, carcass characteristics, blood constituents, immunity and triiodothyronine hormone. Agriculture, 10(4), 138. 10.3390/agriculture10040138

[rda14047-bib-0058] Wang, Z. Y. , Shi, S. R. , Zhou, Q. Y. , Yang, H. M. , Zou, J. M. , Zhang, K. N. , & Han, H. M. (2010). Response of growing goslings to dietary methionine from 28 to 70 days of age. British Poultry Science, 51, 118–121. 10.1080/00071660903431406 20390576

[rda14047-bib-0059] Wu, L. Y. , Fang, Y.‐J. , & Guo, X. Y. (2011). Dietary L‐arginine supplementation beneficially regulates body fat deposition of meat‐type ducks. British Poultry Science, 52, 221–226. 10.1080/00071668.2011.559452 21491245

[rda14047-bib-0060] Wu, Y. , Tang, J. , Cao, J. , Zhang, B. , Chen, Y. , Xie, M. , Zhou, Z. , & Hou, S. (2021). Effect of dietary *L*‐methionine supplementation on growth performance, carcass traits, and plasma parameters of starter pekin ducks at different dietary energy levels. Animals, 11(1), 144. 10.3390/ani11010144 PMC7826553 33440693

[rda14047-bib-0061] Xie, M. , Hou, S. S. , & Huang, W. (2006). Methionine requirements of male white Peking ducks from twenty‐one to forty‐nine days of age. Poultry Science, 85, 743–746. 10.1093/ps/85.4.743 16615358

[rda14047-bib-0062] Zhai, W. , Neuman, S. , Latour, M. A. , & Hester, P. Y. (2007). The effect of dietary L‐carnitine on semen traits of white leghorns. Poultry Science, 86, 2228–2235. 10.1093/ps/86.10.2228 17878454

[rda14047-bib-0063] Zhai, W. , Peebles, E. D. , Wang, X. , Gerard, P. D. , Olanrewaju, H. A. , & Mercier, Y. (2016). Effects of dietary lysine and methionine supplementation on Ross 708 male broilers from 21 to 42 d of age (III): Serum metabolites, hormones, and their relationship with growth performance. Journal of Applied Poultry Research, 25, 223–231. 10.3382/japr/pfw004

